# A Rare Case of Chronic Liver Disease in a Patient Who Previously Presented With Hepatitis E

**DOI:** 10.7759/cureus.35474

**Published:** 2023-02-26

**Authors:** Ramsha Majeed, Mahnoor Khalid, Mahnoor Sherazi, Mohammad J Faisal, Muhammad Awais Waheed

**Affiliations:** 1 Internal Medicine, Fauji Foundation Hospital/Foundation University, Rawalpindi, PAK; 2 Surgery, Fauji Foundation Hospital/Foundation University, Rawalpindi, PAK

**Keywords:** immunocompromised patient, hev genotype, viral hepatitis, chronic liver disease (cld), hepatitis e virus

## Abstract

Hepatitis E virus (HEV) is among the most common causes of acute viral hepatitis. It typically causes acute infection, but some cases of chronic infection have also been recorded. These cases were particularly seen in developed countries, in patients who were immunocompromised, organ transplant recipients, or those with underlying hematological malignancy. However, we encountered a case of hepatitis E presenting as a chronic liver disease in an immunocompetent patient from a developing country. Therefore, more underlying risk factors need to be studied, which may lead to such a rare presentation of hepatitis E.

## Introduction

The hepatitis E virus (HEV) belongs to the Caliciviridae family and has the pathogenicity to cause large outbreaks. It is mostly found in developing countries with substandard sanitation as it spreads by the fecal-oral route. There are four genotypes of HEV [1]. Genotypes 1 and 2 cause acute infections, while genotypes 3 and 4 have a clinically asymptomatic course which can later present as chronic liver disease. However, it is mostly found in immunocompromised patients in developed countries or patients with hematological disorders [2]. Although acute viral infections in developing countries are quite common [3], chronic HEV is rather unheard of. In this case report, we discuss a case of a 61-year-old female who presented with chronic liver disease as sequelae of HEV despite not being immunocompromised, not having a hematological malignancy, and not belonging to a developed country, making it quite a rare presentation. We hope this case report paves the way for further research regarding this rare occurrence.

## Case presentation

A 61-year-old female, a citizen of a rural area in Pakistan, presented to the emergency department with complaints of recurring abdominal pain, which initially started three months ago but had gotten worse in the last two weeks. It was a four out of 10, recurrent, non-radiating pain, with no relieving and aggravating factors. She also complained of abdominal distension for two weeks, associated with progressively increasing swelling of lower limbs. She had experienced lethargy and a disturbed sleep cycle for the last few days. She gave a three months history of gradually worsening jaundice, non-projectile non-bloody vomiting, occasional low-grade fever (documented at 100° F), dark urine, and pruritus. She had a similar history of jaundice seven months back when she tested positive for HEV and received unspecified treatment in another facility.

Her past medical history included a diagnosis of asthma 10 years prior, hypertension two years ago, and an episode of acute coronary disease (stable angina) earlier this year. Past surgical history was significant for cholecystectomy, which was done five years ago. Her medication history revealed she took angiotensin-converting enzyme (ACE) inhibitors for hypertension as well as homeopathic medicine for one month without any improvement of her symptoms. She could not comment on the type of homeopathic medicine she took, and there was no documentation of the medication given. Her social history was positive for hookah smoking which she stopped after being diagnosed with asthma. She denied any history of intravenous drug abuse, alcohol intake, blood transfusion, tattooing, or multiple sexual partners. She had been previously screened sometime in the last five years for HIV, revealing a negative result. She also denied a history of receiving any immunosuppressive therapy, and no hematologic malignancy was noted in the past medical history. 

On physical examination, her vital signs were nearly normal (blood pressure of 125/85 mmHg, respiratory rate of 20 breaths/min, temperature of 97° F) with the exception of a tachycardic pulse (105 beats/minute). Her Glasgow Coma Scale (GCS) was 15/15. Her BMI was 24, which is in the normal range. She was deeply jaundiced and had a distended abdomen. She had a scar mark on her right hypochondrium. The abdomen was slightly tender in the right upper quadrant (RUQ). The liver and spleen were not palpable. She had marked ascites as both fluid thrill and shifting dullness were positive. She also had bilateral pitting pedal edema. There were no other remarkable findings on further examination. Her baseline labs were ordered, which are shown in Table 1.

**Table 1:** Baseline laboratory findings

Investigations	Results (normal values)
White blood cells (WBC) count	7.96 x 109/L (5-10)
Hemoglobin	9.9g/dL (12-16)
Red blood cells (RBC) count	3.22 million cells/mcL (4.2-5.4)
Hematocrit	27.5% (36-42)
Platelet count	160,000 x 109/L (100-400)
Alanine aminotransferase (ALT)	240 U/L (<45)
Aspartate aminotransferase (AST)	250 U/L (<50)
Alkaline phosphatase (ALP)	270 U/L (<300)
Bilirubin	102 µmol/L (2-17)
Albumin	22 g/L (35-50)
Amylase	74 U/L (<140)
Prothrombin time	20 seconds (10-12)
Blood urea nitrogen (BUN9	14 mg/dl (8-23)
Creatinine	0.9 mg/dl (0.7-1.2)
Sodium level	132 mmol/L (136-141)
Potassium level	4.5 mmol/L (3.4-5)
Ferritin	25 ng/mL (10-120)
Glycosylated hemoglobin (HBA1C)	5.5 % (<6.5)
Triglycerides	145 mg/dl (<150)
High-density lipoprotein (HDL)	50 mg/dl (40-60)
Low-density lipoprotein ( LDL)	90 mg/dl (<100)
Thyroid-stimulating hormone (TSH)	2.5 mU/l (0.3-4)
Triiodothyronine (T3)	2.2 nmol/L (1.2-2.8)
Thyroxine (T4)	140 nm/l (77-155)

The patient's lipid profile and thyroid function tests were normal. Serological testing for hepatitis B and C were negative. Other liver pathologies were excluded based on normal autoantibody panel (antinuclear antibody [ANA], anti-mitochondrial antibodies [AMA], smooth muscle antibodies [SMA], liver kidney microsomal antibody [LKMA]), serum ceruloplasmin, iron studies, alpha-1 anti-trypsin levels, and cholangiography. The only serological markers that came back positive were IgM-HEV and IgG-HEV, which along with deranged lab results and clinical findings, raised the suspicion of either reinfection or chronic liver disease due to chronic hepatitis E infection. Due to limited resources in our hospital setting, we were unable to perform genotype testing on the patient. 

**Figure 1:**
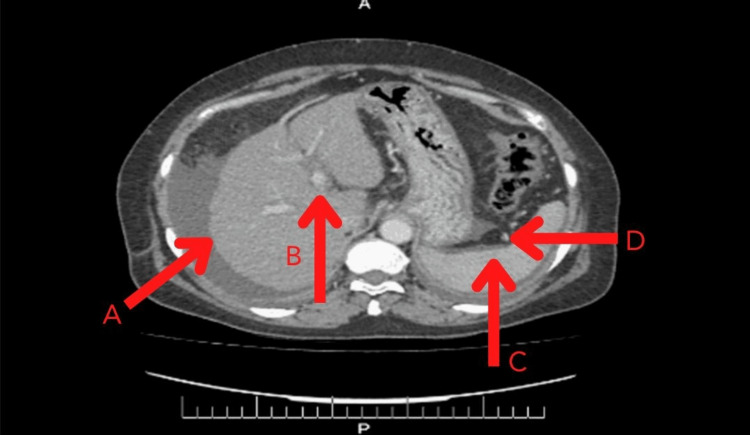
CT of the abdomen and pelvis The liver was subjectively small in size and showed serrated margins (A). The portal vein was mildly dilated and measured approximately 1.3 cm (B). The spleen was mildly enlarged with a splenic index of 550 (C). Few tortuous venous channels were seen at the splenic hilum (splenic varices; D).

**Figure 2:**
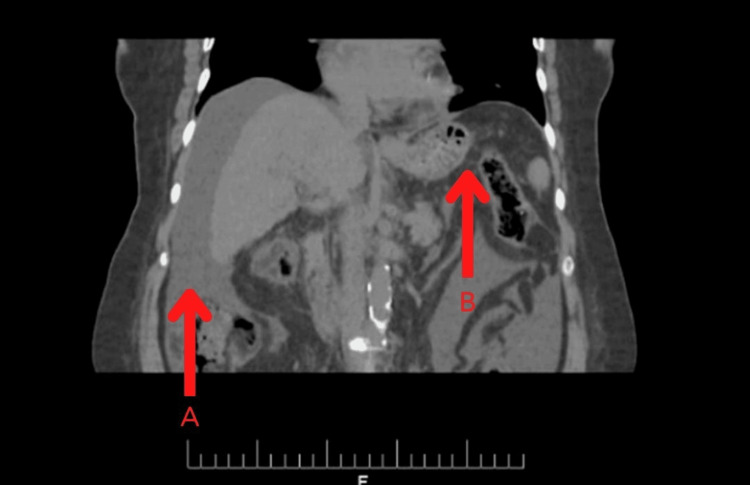
CT of the abdomen Moderate abdominopelvic ascites (A) and fat stranding were seen in the mesentery (B).

Diagnostic paracentesis was performed, and empiric antibiotics were started. Serum albumin ascites gradient (SAAG) was 1.3 g/dL. Chest X-ray and echocardiogram were normal. Ultrasound of the abdomen was ordered, which showed that the liver was shrunken with an irregular margin and coarse parenchymal echotexture. The portal vein was mildly dilated, and mild splenomegaly was also appreciated. Moderate abdominopelvic ascites were also seen. Contrast-enhanced ​​​​​​​CT scan of the abdomen and pelvis also strongly suggested chronic liver disease and portal hypertension, with findings as shown in Figures 1 and 2. The endoscopy performed to screen esophageal varices was insignificant. A liver biopsy was performed, which was remarkable for a portal to central fibrosis consistent with liver cirrhosis. 

Symptomatic treatment along the lines of chronic liver disease was started. The patient was given spironolactone and furosemide for ascites and lower limb swelling. The salt restriction was also advised. The patient was closely monitored for any signs of dehydration, electrolyte abnormality, or worsening hepatic encephalopathy. Ceftriaxone was started empirically because of the suspicion of spontaneous bacterial peritonitis (SBP) and was discontinued after SBP was ruled out. As the patient had initial signs of hepatic encephalopathy, including lethargy and disturbed sleep, the patient was started on syrup lactulose. On one week follow-up, lower limb swelling and ascites had improved; hence furosemide was discontinued. However, her liver function tests were still deranged. Regular follow-up was advised, and the patient was counseled to adhere to her treatment schedule. Screening every six months for hepatocellular carcinoma was also advised.

## Discussion

The vast majority of HEV is known to cause acute illness, accounting for around 20 million cases of HEV in developing countries, with 3.3 million symptomatic cases per year [4]. Although it is among the most common causes of viral hepatitis in the developing world, its propensity to cause acute infection precludes it from being a differential diagnosis when encountering cases of chronic liver disease (CLD). Earlier, HEV was largely known to be endemic to low-middle-income countries where unsatisfactory sanitary conditions were prevalent [5]. It was rarely seen in developed countries, but for the last two decades, cases of chronic infection have been noted in industrialized countries as well. 

Chronic hepatitis E infection has been reported in organ transplant recipients, patients with HIV, cancer patients undergoing chemotherapy or immunotherapy, and those with autoimmune diseases receiving immunosuppressive or immunomodulatory therapy. The first case of chronic hepatitis E was documented in 2008 in an organ transplant recipient infected with genotype 3 of HEV [6]. Studies have shown that about two-thirds of solid organ transplant recipients develop chronic HEV infections. While 60% develop chronic infection, 10% of these progress to cirrhosis within two years. Based on available data, the prevalence of post-transplant HEV infection in non-endemic regions is between 1-2% [7]. In liver transplant patients, particularly, an entity of unexplained chronic hepatitis has been recently encountered in approximately 10% of patients [8]. According to data from the 2011-2018 National Health and Nutrition Examination Survey, a relatively high seroprevalence of HEV (IgG) was recorded among adults with chronic liver disease. It was 8.81% in patients with non-alcoholic fatty liver disease (NAFLD), 6.58% in alcoholic liver disease, 8.66% in hepatitis C virus (HCV), and 19.86% in hepatitis B virus (HBV) patients [9]. A retrospective European cohort study was performed in 2019 to study the burden of HEV in patients with hematological malignancies. The results reflected progression to chronic hepatitis in 34% of the patients overall [10]. In HIV patients, only two cases of chronic HEV were reported until 2013; however, many more have been encountered since then, especially in patients with severe immunosuppression [11]*. *However, information on chronic cases of cancer and autoimmune disease patients remains limited, and more research is needed to fully understand their epidemiology [12]. 

We encountered a case of HEV from a rural area, which was not linked to any of the above-mentioned conditions. Hepatitis E genotypes 1 and 2 are mostly found in developing nations of the world. In contrast, genotypes 3 and 4 are found in developed nations [13]. The most important cause is contaminated water or food sources [14]. The presence of fecal matter in water and undercooked pork are the main culprits. Overcrowded areas also pose a risk, with people easily transmitting the virus due to an unhygienic lifestyle. Less common causes may include blood transfusions, and infected pregnant women may pass HEV on to the fetus resulting in neonatal mortality and morbidity, but this has received very little attention, and more research is required before it can be proven [15]. The patient we discuss in this case report comes from a rural background with unsatisfactory hygienic conditions, possibly being the source of her infection. However, due to limited resources in our region, it was not possible to perform genotype testing on this patient.

The symptoms of HEV appear 15-60 days after exposure and can vary from person to person, with some people not experiencing any symptoms at all. Possible symptoms include loss of appetite, fever, nausea, vomiting, fatigue, pruritus, jaundice, clay-colored stools, dark urine, and pain in the upper abdomen, especially over the liver [16]. There are currently no specific tests for the diagnosis of the disease, so a patient history that covers all possible causes and physical examination is extremely important in reaching a diagnosis. Blood and/or stool tests are used to detect the presence of certain antibodies against the virus. Blood tests showing raised bilirubin and liver enzymes and decreased leukocytes are also indicative of an HEV infection [17]. There is no specific treatment for an acute HEV infection, as most cases spontaneously resolve. Avoiding alcohol, eating a balanced diet, drinking plenty of fluids, and resting are advised. Although clinical data is lacking regarding the use of antiviral therapy (ribavirin), it has been shown to reduce the course of the illness and decrease overall morbidity in a couple of clinical trials/case reports [18]. However,15% of patients fail to reach full recovery and turn into chronic cases. For some rare cases, a liver transplant may be indicated if the patient fulfills the criteria required for transplantation [18]. 

Although HEV often resolves on its own, some severe chronic cases can lead to inflammation and damage to the liver, causing serious complications. The pathogenesis of this mechanism is poorly understood. However, a chronic HEV infection is defined as the persistence of IgG and IgM and liver inflammation lasting for three to six months [7]. On performing an ultrasound on this patient, similar findings of chronic inflammation and damage were seen. This ruled out reinfection as a cause and reinforced the suspicion of HEV presenting as chronic liver disease. This was also supported by the patient's serology showing positive IgG and IgM on her visit three months later. 

Our patient was diagnosed with CLD due to chronic hepatitis E infection. Such cases are rare to find in the medical literature as hepatitis E has historically been associated with acute infection rather than a chronic one. This possibility should be investigated further with the help of further research. 

## Conclusions

HEV, most commonly transmitted via the fecal-oral route, happens to be the most common form of viral hepatitis. Although there has been adequate emerging data on HEV presenting as chronic liver disease in the past few years, chronically infected HEV patients in developing countries are still unusual. The existing literature on the correlation between HEV and CLD in immunocompetent patients of developing countries is lacking. Although more research must be completed before a causative relationship can be suggested, this case report is an encouraging step forward. A series of laboratory tests, multiple imaging studies, and invasive procedures, including endoscopy and liver biopsy, all failed to provide an alternative diagnosis. Thus, we believe that in developing countries where HEV incidence is high, it may be helpful to include serological markers for hepatitis E in the assessment of CLD patients for whom a cause cannot be determined.

## References

[1] Horvatits T, Schulze Zur Wiesch J, Lütgehetmann M, Lohse AW, Pischke S (2019). The clinical perspective on hepatitis E. Viruses.

[2] de Niet A, Zaaijer HL, ten Berge I, Weegink CJ, Reesink HW, Beuers U (2012). Chronic hepatitis E after solid organ transplantation. Neth J Med.

[3] Aslan AT, Balaban HY (2020). Hepatitis E virus: epidemiology, diagnosis, clinical manifestations, and treatment. World J Gastroenterol.

[4] Kamar N, Pischke S (2019). Acute and persistent hepatitis E virus genotype 3 and 4 infection: clinical features, pathogenesis, and treatment. Cold Spring Harb Perspect Med.

[5] (2020). Hepatitis E questions and answers for health professionals. https://www.cdc.gov/hepatitis/hev/hevfaq.htm#:~:text=years%20of%20age).-,Where%20is%20hepatitis%20E%20most%20common%3F,supply%20and%.

[6] Kamar N, Garrouste C, Haagsma EB (2011). Factors associated with chronic hepatitis in patients with hepatitis E virus infection who have received solid organ transplants. Gastroenterology.

[7] Murali AR, Kotwal V, Chawla S (2015). Chronic hepatitis E: a brief review. World J Hepatol.

[8] Haagsma EB, van den Berg AP, Porte RJ, Benne CA, Vennema H, Reimerink JH, Koopmans MP (2008). Chronic hepatitis E virus infection in liver transplant recipients. Liver Transpl.

[9] Wong RJ, Cheung R, Gish RG, Chitnis AS (2021). Prevalence of hepatitis E infection among adults with concurrent chronic liver disease. J Viral Hepat.

[10] von Felden J, Alric L, Pischke S (2019). The burden of hepatitis E among patients with haematological malignancies: a retrospective European cohort study. J Hepatol.

[11] Rivero-Juarez A, Lopez-Lopez P, Frias M, Rivero A (2019). Hepatitis E infection in HIV-infected patients. Front Microbiol.

[12] Kamar N, Izopet J, Pavio N, Aggarwal R, Labrique A, Wedemeyer H, Dalton HR (2017). Hepatitis E virus infection. Nat Rev Dis Primers.

[13] Kamar N, Selves J, Mansuy JM (2008). Hepatitis E virus and chronic hepatitis in organ-transplant recipients. N Engl J Med.

[14] Ma Z, de Man RA, Kamar N, Pan Q (2022). Chronic hepatitis E: advancing research and patient care. J Hepatol.

[15] Krain LJ, Atwell JE, Nelson KE, Labrique AB (2014). Fetal and neonatal health consequences of vertically transmitted hepatitis E virus infection. Am J Trop Med Hyg.

[16] Xin S, Xiao L (2016). Clinical manifestations of hepatitis E. Adv Exp Med Biol.

[17] Hepatitis A and E. https://www.hopkinsmedicine.org/health/conditions-and-diseases/hepatitis/hepatitis-a.

[18] Shah S, Cannon M (2018). Hepatitis E virus infection: signs, symptoms and management. Pharm J.

